# Philadelphia Medicines for Egypt and Italy

**Published:** 1877-07-20

**Authors:** 


PHILADELPHIA MEDICINES FOR EGYPT AND ITALY.—The Philadelphia *Ledger* says Messrs. Wm. R. Warner & Co., of that city, manufacturing chemists, recently furnished the medical department of the Egyptian army with a large supply of sugar-coated pills, for use in the army, and Dr. Edward Warren, Bey, Surgeon-in-Chief, wrote that the pills were "Portable, indestructible, and yet most potent in their operation; they were easily and safely carried throughout every portion of Northern and Equatorial Africa." The same firm have just received an order by cable for two hundred thousand quinine dragees (sugar-quoted pills) for use in one of the large government hospitals in Rome, Italy.

